# Investigating the transition of psychopathological symptoms from childhood to adolescence in maltreated youth: a cross sectional study

**DOI:** 10.3389/fpsyt.2025.1513100

**Published:** 2025-06-19

**Authors:** Elisa Fucà, Veronica Sperandini, Stefania Falvo, Paola De Rose, Stefano Vicari

**Affiliations:** ^1^ Child and Adolescent Neuropsychiatry Unit, Bambino Gesù Children’s Hospital, IRCCS, Rome, Italy; ^2^ Department of Life Science and Public Health, Catholic University of the Sacred Heart, Rome, Italy

**Keywords:** maltreatment, neglect, child behavior checklist, adaptive skills, child mental health

## Abstract

**Inrtroduction:**

The transition to adolescence is a crucial developmental phase in which notable and significant changes in behavior may emerge. Despite child maltreatment represents one of the most critical public health risk factors for mental health disorders, poor is known about possible differences in psychopathological symptoms between children and adolescents who experienced maltreatment. Using a cross-sectional, multi informant approach, this study had three objectives: (i) to examine age-related differences in psychopathological symptoms in maltreated children and adolescents using a multi-informant, cross-sectional approach, (ii) to investigate sex differences in psychopathological symptoms across age groups, and (iii) to assess differences in adaptive skills based on age and sex.

**Methods:**

One hundred and ninety-five youth with a history of maltreatment (6–17 years) were included. Psychopathological symptoms were assessed by caregiver- reports, self-reports and by a clinician’s rating scale, whereas adaptive skills were assessed by the Adaptive Behavior Assessment System.

**Results:**

Parent-reports and evaluation of adaptive skills highlighted a worse social functioning in adolescents in comparison with children. These findings were corroborated by self-reports. Both parent- and self-reports, but not clinician’s ratings, revealed some sex differences, with school-age girls exhibiting more parent-reported somatic complaints and male adolescents exhibiting more parent-reported aggressive behaviors. Moreover, female adolescents displayed more self-perceived ineffectiveness and interpersonal difficulties.

**Discussion:**

Overall, these findings indicate deficits in adaptive functioning within the social domain of maltreated children and adolescents, highlighting the critical need for a deeper exploration of these impairments, as disrupted social functioning during adolescence may further impede their development and integration into society. These findings underscore the need for targeted clinical interventions to address the worsening social adaptation in this population.

## 1 Introduction

Psychopathology is a neurodevelopmental process that begins early in life and continues to evolve throughout the lifespan (1). Indeed, it is well-established that children exhibiting psychopathological symptoms during childhood and adolescence are more likely to experience similar issues in adulthood (2–4). Examining the stability and evolution of children’s behavior over time can help differentiate between temporary developmental phases and those that indicate enduring psychopathological conditions. While for some children internalizing and externalizing problems may represent a transitory phase of development (5), psychopathological symptoms can also take different forms depending on the developmental phase. In particular, the transition to adolescence represents a crucial developmental phase in which notable and significant changes in behavior may emerge (6). Indeed, adolescence is marked by significant physical changes due to puberty, including sexual maturation, and by significant changes in brain development, especially in the maturation of the frontal lobe and fronto-limbic regions (7). These changes in brain structure are associated with significant changes in some cognitive skills, such as cognitive control and self-regulation (8). Societal changes also significantly impact adolescents as they assume more adult roles. Social interactions and affiliation with peers take on particular significance with a shift of their primary source of emotional support from parents to peers (9). Considering these changes, it is reasonable to expect a variation in the clinical manifestations of psychopathological symptoms in the transition from childhood to adolescence. A review on this topic showed that, as children progress into adolescence, rates of depression, panic disorder, agoraphobia, and substance use disorders rise, while separation anxiety disorder and attention-deficit hyperactivity disorder (ADHD) decrease. In the transition from adolescence to early adulthood, panic disorder, agoraphobia, and substance use disorders continue to increase, with further reductions in separation anxiety disorder and ADHD. Additionally, other phobias and disruptive behavior disorders also decline (10). A more recent longitudinal study confirmed these findings, reporting that, in childhood, issues with hyperactivity/impulsivity, motor coordination, and conduct were prevalent, whereas adolescents tended to exhibit difficulties with emotional regulation, anxiety, and attention (11). It should be emphasized, however, that the interplay between emotionality and risk-taking can also yield positive outcomes, as adolescents may be driven by emotional forces that promote prosocial behaviors, including academic and family-oriented pursuits (12, 13). Consequently, adolescence has increasingly been recognized as a phase of both vulnerability and opportunity (6), shaped by the capacity of affective systems to guide behavior.

It is also essential to consider discrepancies between parent reports and self-reports, as these differences can provide a more comprehensive understanding of the psychopathological changes that accompany adolescence. For instance, previous literature documented not only that poor agreement between parent and self-report can be detected for conduct problems and ADHD, but also that parents seem to be much more likely to identify the externalizing problems than internalizing problems in their children when their children did not (14). Therefore, assessing psychopathological symptoms using both parent reports and self-reports may be crucial. This dual approach allows for a more accurate and comprehensive understanding of their emotional and behavioral difficulties, capturing both externalizing and internalizing problems that may otherwise go unnoticed.

Mental health outcomes in pediatric age should be considered taking into account sex differences. Indeed, it has been documented that, in pediatric age, externalizing disorders are more frequently observed in males than females, whilst the reverse pattern is true for internalizing disorders (15–17). However, a study conducted on 11,384 participants found that boys aged 9–10 exhibit higher levels of parent-reported psychopathology compared to girls and that boys exhibited greater scores and a higher likelihood of clinically elevated scores in areas such as withdrawn/depressed behaviors, attention problems, aggressive behaviors, and social difficulties (18). Girls, on the other hand, did not show higher scores or more frequent clinical elevations in any syndrome, including anxiety or depression. As puberty progressed, both boys and girls were more likely to display clinically elevated syndrome scores (18). All these studies emphasize the importance of investigating sex differences in psychopathological symptoms in the transition to adolescence.

Child maltreatment, encompassing physical, sexual, and emotional abuse, neglect, and exposure to intimate partner violence, represents one of the most critical but preventable public health risk factors for mental health disorders (19, 20). Individuals exposed to abuse or neglect during childhood, whether as children or adults, face an increased risk of various negative mental health outcomes, including internalizing and externalizing psychopathology, posttraumatic stress disorder (PTSD), psychotic symptoms, and personality disorders (21–25). A recent meta-analysis found that more than 1.8 million cases of depressive, anxiety, and substance use disorders could be prevented if childhood maltreatment was eradicated (26). Focusing on pediatric age, a recent systematic review confirmed the significant correlation between childhood emotional maltreatment and adolescent psychopathology (27). Moreover, children who experienced maltreatment often show delays in the development of adaptive skills (28), which are crucial for coping with daily challenges. Understanding how these abilities evolve during the transition from childhood to adolescence is essential, as this period marks a critical phase for social, emotional, and cognitive development, particularly in vulnerable populations.

However, despite the significant research interest in mental health outcomes in maltreated children and adolescents, poor is known about possible differences in psychopathological symptoms between children and adolescents who experienced maltreatment. Since childhood maltreatment is an adverse experience that lead to abnormal developmental trajectory (29), it cannot be assumed that psychopathological changes associated with the transition to adolescence in maltreated youth follow the same trends observed in non-maltreated youth. Understanding how behavioral problems in maltreated children manifest at various stages of development, and especially how these problems may change in the transition into adolescence, is crucial for identifying early interventions that could mitigate long-term mental health risks and promote healthier developmental outcomes during this critical period.

Therefore, the current study had three main goals: i) to explore age group differences in psychopathological symptoms in children and adolescents who experienced maltreatment using a multi-informant, cross-sectional approach, ii) to explore sex differences in psychopathological symptoms across different age groups, and iii) to explore the differences in adaptive skills across age and sex groups.

Our study aimed to address key gaps in the existing literature by examining sex-specific differences in the association between maltreatment and psychopathological symptoms across different developmental stages. While previous research has documented general links between maltreatment and mental health outcomes, few studies have simultaneously considered sex differences, multiple informants (parents and clinicians), and distinct symptom profiles. By integrating these factors, our work provides a more nuanced understanding of how maltreatment manifests differently in boys and girls, offering insights into potential sex-specific pathways of risk.

## 2 Materials and methods

### 2.1 Participants

One hundred and ninety-five children and adolescents with a history of maltreatment (physical maltreatment; exposure to intimate partner violence or neglect) ranging in age from 6 to 17 years were included in the study. We distinguished among participants aged 6 to 11 (school-age children: N=100); and participants aged 12 to 17 (adolescents: N=95). **Table 1** summarizes the demographic characteristics of the sample. The two age groups were matched for sex (*p* =0.095) and IQ (*p* = 0.469). Selection criteria included the history of a maltreatment substantiated by social services and/or judicial authorities and the age ranging between 6 and 18 years. Exclusion criteria were age <6 or >17.11 years; the ascertained presence of neurological conditions, such as epilepsy; language barrier hampering questionnaire compilation by caregivers.

**Table 1:** Demographic characteristics of the sample.

	Total sample N=195	School-age children N=100	Adolescents N=95
Age	11.79 ± 3.14	9.14 ± 1.59	14.59 ± 1.52
Sex (M/F; %)	47.2/52.8	47/53	41/59
IQ	102.53 ± 18.45	103.5 ± 18	101.5 ± 18.9

### 2.2 Procedure

This was a retrospective, cross-sectional study; data were collected from a file review of a database including data from 360 youth who experienced a form of maltreatment and referred for a clinical evaluation for behavioral/psychopathological issues at the Child and Adolescent Neuropsychiatry Unit of a pediatric hospital between January 2018 and April 2024. The clinical assessment took place over two or three half-days on a day hospital basis. The participants underwent neuropsychiatric, neuropsychological, and psychopathological evaluations conducted by a team that included a child neuropsychiatrist and psychologists with expertise in child psychopathology. During the day hospital visit, caregivers were asked to complete questionnaires regarding their child’s mental health, adaptive functioning, and overall functioning. Due to the retrospective design, data were collected from the hospital records and clinic charts and the de-identified data were analyzed.

#### 2.2.1 Compliance with ethical standards

All caregivers signed a written informed consent for data use for research purposes and a privacy statement that ensures that data will be kept confidential. The study was conducted according to the guidelines of the Declaration of Helsinki and was approved by the local Ethical Committee (practice n°3188/2023, prot. N.827, NPI 3-03-2023).

### 2.3 Measures

#### 2.3.1 Intelligence quotient

Cognitive development was assessed by Wechsler Intelligence Scale for Children (WISC-IV) (30). The instrument is made of e 10 core subtests, namely Block Design, Similarities, Digit Span, Picture Concepts, Coding, Vocabulary, Letter–Number Sequencing, Matrix Reasoning, Comprehension and Symbol Search. WISC-IV administration provides four different indexes: Verbal Comprehension Index, Perceptual Reasoning Index, Working Memory Index, and Processing Speed Index. In cases of language problems, we administered non-verbal instruments. In particular, we used the Leiter International Performance Scale – 3rd Edition - Leiter-3 (31) – which provides a nonverbal measure of intelligence and assesses the ability to reason by analogy, by matching and perceptual reasoning in general, irrespective of language and formal schooling. The Global Non-Verbal Intelligent Quotient obtained through this test is based on four subtests: Figure Ground, Form Completion, Classification and Analogies, and Sequential Order. We used also the Colored (32) or Standard Progressive Matrices (29), to assess mental ability associated with abstract reasoning, and considered a nonverbal estimate of fluid intelligence. The test consists of increasingly difficult pattern matching tasks and has little dependency on language abilities.

#### 2.3.2 Adaptive functioning

Adaptive functioning was assessed using the Adaptive Behavior Assessment System-Second Edition Parent Form 5-21 (ABAS II) (33). The ABAS-II 5–21 is a parent-report questionnaire for caregivers of individuals aged 5–21 years. ABAS-II 5–21 yields three specific domain scores (Conceptual, Social, and Practical) and an overall General Adaptive Composite (GAC).

#### 2.3.3 Psychopathological assessment

The psychopathological assessment included multi-informant data (caregiver-reported, child-reported, and clinicians’ observation).

Child Behavior Checklist (CBCL). The CBCL is a parent/caregiver report form to screen for emotional, behavioral, and social problems (34). The school-age version (CBCL/6–18) is for children aged 6 to 18 years. It is composed of eight Empirically Based Syndromes Scales, three general domains, six DSM-oriented scales, and three 2007 Scale Scores: Sluggish Cognitive Tempo, Obsessive-Compulsive, Post-traumatic Stress Problems. For the current study, Empirically Based Syndromes Scales (Anxious/Depressed, Withdrawn/Depressed, Somatic Complaints, Social Problems, Thought Problems, Attention Problems, Rule-Breaking Behavior and Aggressive Behavior) were considered.

The Child Depression Inventory, 2nd edition (CDI-2), Italian standardized version. The CDI-2 is completed by the child and assesses age-specific manifestations of depressive symptoms (35). The CDI-2 uses a normative sample to produce standard T scores in order to compare the raw scores to the average population scores (matched to age and sex). The instrument consists of 28 items divided into two mains scale and subscales. The first main scale, titled Emotional Problems scale, includes Negative Mood/Physical Symptoms and Negative Self-Esteem, and component items evaluate symptoms of distress, such as sadness, guilt, self-loathing, and anomalies in sleep patterns, eating habits, and energy levels. The second main scale, titled Functional Problems Scale, includes Ineffectiveness and Interpersonal Problems, and component items indicate inhibited social relationships, such as peer and family relationships and maladjustment in school. In the current study, Data from CDI-2 were available for a subsample of 121 participants (school-age group, N= 61; adolescents, N = 60).

Functional impairment was measured using the C-GAS, a widely used clinician-rating tool designed to measure the lowest level of functioning of a child or adolescent within the last 30 days. It ranges from 1 to 100, where 1 represents children with the most functional impairments and 100 represents children with the highest functional adaptability (36).

#### 2.3.4 Data analysis

Descriptive statistics were used to analyze demographic characteristics. T-tests, multivariate analysis of variance (ANOVA) and multivariate analysis of covariance (ANCOVA) were computed to detect differences between age and sex groups; for the ANCOVA, the scores of the Post-traumatic Stress Problems scale as covariate to control for potential confounding factors associated with the presence of symptoms of PTSD as reported by parents. Statistical tests were used with a significance level of *p* < 0.05. The missing data have not been replaced. Statistical analyses were performed using the Statistical Package for the Social Sciences, version 13.0 (IBM Corp, Armonk, NY, USA). Missing data from the CDI-2 were not replaced.

## 3 Results

### 3.1 Differences on caregiver-report psychopathological symptoms

#### 3.1.1 Differences between age groups

Multivariate ANCOVA on CBCL scores revealed that adolescents exhibited significantly worse scores than school-age children at the Social Problems scale (resisted to Fisher *post-hoc* correction). Results are summarized in **Table 2**.

**Table 2:** Differences in CBCL scores between school-age children and adolescents.

CBCL scale	School-age children N=100	Adolescents N=95	F	*p*	Partial η^2^
Anxious/Depressed	63.5 ± 10.66	65.86 ± 11.53	.265	0.608	0.001
Withdrawn/Depressed	62.95 ± 10.15	65.27 ± 9.54	.064	0.801	<0.001
Somatic Complaints	61.42 ± 9	63.18 ± 9.21	.007	0.933	<0.001
Social Problems	61.34 ± 9.77	65.61 ± 10.51	4.299	0.039*	0.022
Thought Problems	62.21 ± 10.47	65.46 ± 9.98	1.007	0.317	0.005
Attention Problems	62.1 ± 9.97	64.42 ± 10.27	.055	0.815	<0.001
Rule-Breaking Behavior	59.91 ± 8.57	62 ± 9.05	.550	0.459	0.003
Aggressive Behavior	62.77 ± 11.04	65.38 ± 11.83	.068	0.795	<0.001

*p<0.05.

#### 3.1.2 Sex differences

ANCOVA revealed that, among school-age children, females exhibited slightly higher scores at the Anxious/Depressed scale and significantly higher scores than males at the Somatic Complaints scale; of note, these differences did not resist to Fisher *post-hoc* correction. Results are summarized in **Table 3**.

**Table 3:** Sex differences in CBCL scores among school-age children.

CBCL scale	Males N=47	Females N=53	F	*p*	Partial η^2^
Anxious/Depressed	62.9 ± 11.18	64.17 ± 10.12	3.791	0.054	0.038
Withdrawn/Depressed	63.73 ± 10.73	62.06 ± 9.5	.427	0.515	0.004
Somatic Complaints	60.58 ± 9.49	62.36 ± 8.46	4.653	0.033*	0.046
Social Problems	62.19 ± 11	60.38 ± 8.07	.601	0.44	0.006
Thought Problems	62.49 ± 10.99	61.89 ± 9.33	.028	0. 869	<0.001
Attention Problems	62.68 ± 10.55	61.42 ± 9.34	.140	0.71	0.001
Rule-Breaking Behavior	60.72 ± 9.3	59 ± 7.65	.728	0.396	0.007
Aggressive Behavior	64.3 ± 12.12	61.04 ± 9.51	2.618	0.109	0.026

*p<0.05.

Among adolescents, males exhibited significantly higher scores than females at the Aggressive Behavior scale (resisted to Fisher *post-hoc* correction). Results are summarized in **Table 4**.

**Table 4:** Sex differences in CBCL scores among adolescents.

CBCL scale	Males N=39	Females N=56	F	*p*	Partial η^2^
Anxious/Depressed	66.53 ± 11.36	65.39 ± 11.73	.002	0.969	<0.001
Withdrawn/Depressed	66.26 ± 10.9	64.59 ± 8.5	.423	0.517	0.005
Somatic Complaints	62.41 ± 11.35	64.82 ± 9.92	1.140	0.289	0.012
Social Problems	66.74 ± 18.71	55.81 ± 11.94	.512	0.476	0.006
Thought Problems	66.87 ± 10.22	64.48 ± 9.77	1.291	0. 259	0.014
Attention Problems	66.1 ± 11.31	63.27 ± 9.4	1.788	0.184	0.019
Rule-Breaking Behavior	63.56 ± 9.71	61.02 ± 8.5	1.573	0.213	0.017
Aggressive Behavior	69.49 ± 13.5	62.52 ± 9.65	11.381	0.001*	0.11

*p<0.05.

### 3.2 Differences on self-report psychopathological symptoms

#### 3.2.1 Differences between age groups

The analysis of CDI-2 scores revealed that children exhibited slightly lower scores than adolescents at the Emotional Problems scale, with differences approaching the statistical significance (54.51 ± 13.1 and 59.2 ± 13.62 respectively; *p* = 0.055; Cohen’s *d* = 0.35). Moreover, children exhibited significantly lower scores than adolescents at the Functional Problems Scale (54.78 ± 11.2 and 61.42 ± 12.47 respectively; *p* = 0.002; Cohen’s *d* = 0.56).

#### 3.2.2 Sex differences

Among school-age children, sex differences did not emerge for Emotional Problems Scale (54.59 ± 12.2 and 54.41 ± 14.22 for males and females, respectively; *p* = 0.958; Cohen’s *d* = 0.014) nor for Functional Problems Scale (54.72 ± 11.87 and 54.86 ± 10.6 for males and females, respectively; *p* = 0.962; Cohen’s *d* = 0.012). On the other hand, among adolescents, females exhibited significantly higher scores than males at the Functional Problems Scale (64.1 ± 12.29 and 56.87 ± 11.68 respectively; *p* = 0.026; Cohen’s *d* = 0.6), but not at the Emotional Problems scale (59.54 ± 14.35 and 58.59 ± 12.54 respectively; *p* = 0.797; Cohen’s *d* = 0.22).

### 3.3 Differences on clinicians’ ratings

#### 3.3.1 Differences between age groups

In order to detect possible differences on clinicians rating, we compared the age groups on C-GAS scores. T-test revealed that school-age children exhibited slightly higher C-GAS scores, but this difference did not reach the statistical significance (56.39 ± 8.95 and 54.16 ± 8.56 respectively; *p* = 0.07; Cohen’s *d = 0.26*).

#### 3.3.2 Sex differences

T-tests were performed to explore sex differences on C-GAS scores within the age groups, but we did not detect significant differences for school-age participants (56.3 ± 10.26 and 56.49 ± 7.3 for males and females, respectively; *p* = 0.917; Cohen’s *d* = 0.32) nor for adolescents (55.31 ± 9.97 and 53.36 ± 7.42 for males and females, respectively; *p* = 0.277; Cohen’s *d =* 0.0007).

### 3.4 Differences on adaptive skills

#### 3.4.1 Differences between age groups

Multivariate ANOVA on ABAS II scores showed that adolescents exhibited significantly worse scores than children in the social domain (77.3 ± 19.13 and 83.35 ± 17.76 respectively; *p* = 0.024; partial η^2^ = 0.026; resisted to Fisher *post-hoc* correction), but not in the conceptual (82.53 ± 20.27 and 82.65 ± 18.52 respectively; *p* = 0.966; partial η^2^ = 0.001) nor in the practical domains (83.34 ± 17.67 and 87 ± 17.14 respectively; *p* = 0.143; partial η^2^ = 0.011).

#### 3.4.2 Sex differences

Among school-age participants, females exhibited slightly higher scores than males in the social domain of the ABAS-II (86.74 ± 16.61 and 80.41 ± 18.35 respectively; *p* = 0.077; partial η^2^ = 0.032). No sex differences emerged for the conceptual (84.61 ± 17.19 and 80.94 ± 19.6 respectively; *p* = 0.329; partial η^2^ = 0.01) or the practical domains (89.84 ± 17.76 and 84.6 ± 17.57 respectively; *p* = 0.130; partial η^2^ = 0.024). Among adolescents, females exhibited significantly higher scores than males in both conceptual (87.22 ± 20.25 and 75.74 ± 18.53 respectively; *p* = 0.007; partial η^2^ = 0.078; resisted to Fisher *post-hoc* correction) and practical domains (87.05 ± 15.8 and 77.97 ± 19 respectively; *p* = 0.014; partial η^2^ = 0.064; resisted to Fisher *post-hoc* correction). Conversely, no sex differences emerged for the social domain (79.65 ± 19.77 and 73.89 ± 17.87 respectively; *p* = 0.155; partial η^2^ = 0.022).

## 4 Discussion

The first aim of the current research was to explore age group differences in psychopathological symptoms in children and adolescents who experienced maltreatment using a multi-informant, cross-sectional approach. Overall, adolescents exhibited more difficulties in social relationships – both parent and self-reported – and slightly worse global functioning than children.

Concerning parent-reports, adolescents exhibited higher scores at the Social Problems scale of the CBCL. This scale covers various difficulties that children and adolescents may face in their relationships with others, for example being teased, disliked, or overly dependent on others. Our results are consistent with previous literature indicating that the negative effects of maltreatment on children’s social functioning include difficulties in peer relationships, and in social perspective-taking skills, reduced interactive play, impaired emotional regulation, and shortcomings in self-discipline (37–41). Given that most previous studies focused on specific age ranges or considered broad age ranges without distinguishing between children and adolescents, the current research confirms and extends these findings, providing preliminary evidence that the transition to adolescence plays a crucial role in the clinical manifestation of the impairment of social functioning in maltreated children. The differences between children and adolescents on social problems could be accounted, at least in part, by alterations in emotion regulation throughout childhood and adolescence. For instance, a cross-sectional study conducted on a sample of 1397 participants and investigating the changes in emotion regulation throughout childhood and adolescence documented reduced use of adaptive strategies and increased use of maladaptive strategies in participants between 12 and 15 years old compared with younger or older participants (42).

The results from parent reports were corroborated by self-reports. Specifically, the analysis of the CDI-2 revealed that adolescents reported significantly higher scores on the Functional Problems Scale, which includes items reflecting issues such as impaired social relationships, difficulties with peers and family, and maladjustment in school. This suggests that maltreated adolescents tend to exhibit awareness of their difficulties in social functioning. This is in line with previous research suggesting that, among population exposed to sexual abuse, adolescents compared to children show more pervasive psychological distress, exhibiting, for instance, higher levels of depressive symptoms, greater negative reactions by others, lower self-worth, and less social support than children (43). Finally, clinicians’ ratings revealed that adolescents tended to exhibit slightly worse functioning than children. Since social skill difficulties may increase the likelihood of maltreated individuals developing behavioral issues, such as peer rejection, behavioral maladjustment, conduct disorders (44–47), we can hypothesize that adolescents with a history of maltreatment may be particularly vulnerable to more severe behavioral and emotional difficulties compared to children, as their developing social skills may exacerbate challenges in peer relationships and increase the risk of maladjustment and conduct-related problems. This could explain the slightly worse functioning observed in adolescents, potentially indicating that social skill deficits have a compounding effect as individuals grow older. Moreover, the observed differences may be also attributed to the fact that adolescents were exposed to maltreatment for a longer period, which likely amplified its negative effects on their social abilities.

The second goal of this work was to explore sex differences in psychopathological symptoms across age groups. Altogether, our data on sex differences suggest that school-age girls who experienced maltreatment tend to exhibit more parent-reported somatic complaints, whereas, among adolescents, boys tend to exhibit more parent-reported aggressive behaviors. Moreover, female adolescents displayed more self-perceived ineffectiveness and interpersonal difficulties. Clinicians’ ratings did not detect significant sex differences in the overall functioning nor in children nor in adolescents, with both groups exhibiting mild level of impairment. Specifically, the analysis of parent reports revealed that, among children, females reported more somatic complaints than males. The “Somatic Complaints” scale of the CBCL includes items associated with different forms of somatic problems, such as headaches, nausea, skin problems. The presence of somatic problems in maltreated children is consistent with previous reports on the association between the exposure to psychological and sexual abuse (48–50). Moreover, a study conducted in the pediatric emergence department of a university hospital found that among the 245 patients managed for child abuse and neglect the 31% was admitted for somatic complaints (51), suggesting that physical symptoms may often be a manifestation of underlying abuse or neglect. Despite the fact that comparisons did not resist to *post-hoc* analysis, our findings about the higher scores obtained by females on the Somatic Complaints scale are consistent with those reported by Lamers-Winkelman and colleagues (2012) in a sample of 275 child exposed to intimate partner violence (6–12 years of age) (52). The authors, using the CBCL, found that girls more often had stomach aches than boys. The results of the present study further highlight the importance of thorough assessments in pediatric healthcare settings to identify potential cases of maltreatment. Considering also the results of self-reports, our findings can be interpreted in the light of previous findings in adult population, suggesting that somatization may predict subsequent self-reported symptoms of depressed mood, but only in women (53).

On the other hand, caregivers of male adolescents reported significantly higher levels of aggressive behaviors compared to those of female adolescents. Previous literature about sex differences on aggressive behaviors in young individuals exposed to maltreatment provided contrasting results. Indeed, some authors reported no gender differences in the longitudinal associations among childhood maltreatment and aggression (54), whereas other studies documented that males seem to be more prone to engage in aggression compared with females through the pathway of irritability (55). Intriguingly, the study conducted in a sample of 1797 adolescents aged 14–16 revealed that abuse and neglect experienced during adolescence can affect depressive symptoms and aggression differently, depending on sex (56). Specifically, the authors found that experiencing abuse reduced the increase rate of aggression over time in girls, whereas neglect increased the initial value of aggression only in boys (56). These findings suggest that the relationship between maltreatment and aggression may differ by sex, with potential variations in the type of maltreatment and its impact on aggressive behaviors. The results of the present study, which indicate higher levels of aggression reported in male adolescents, align with research suggesting that boys may be more susceptible to aggression through certain pathways, such as irritability (55). However, the observed differences also highlight the complexity of these associations, suggesting that different forms of maltreatment, such as abuse or neglect, may uniquely shape the developmental trajectories of aggression in boys and girls. Further research is needed to explore these sex-specific pathways and to clarify the mechanisms underlying the contrasting results in the literature. It should be pointed out that our findings are partially inconsistent with previous research suggesting comparable prevalence of emotional problems between boys and girls exposed to physical abuse (57). This discrepancy should be explained by considering that we did not take into account specific differences between different forms of maltreatment/abuse in our sample.

The analysis of self-reports failed to detect any sex difference among school-age children. This is consistent with previous findings reporting no significant sex main effects regarding social self-efficacy in a sample of 305 maltreated children ages 5 to 12 year (58). Conversely, among adolescents, girls scored higher at the Functional Problems Scale of the CDI-2, indicating more ineffectiveness and interpersonal problems. These sex differences in internalizing symptoms align with previous reports on the higher vulnerability of maltreated girls to developing internalizing symptoms (15–17). These results also align with literature reporting not only that childhood maltreatment negatively relates with self-compassion (59) but also that self-compassion moderates the effect of self-criticism in the link between childhood maltreatment and psychopathology (60). Since previous findings indicated that older girls exhibit lowest self-compassion levels compared to younger females or all-age males and that self-compassion seems to have a greater protective effect on anxiety for boys than for girls (61), the most prominent proneness of maltreated female adolescents to internalizing symptoms is not surprising. The observed sex differences may be also explained by sex-specific neurobiological patterns associated with maltreatment. Indeed, literature on trauma-associated psychopathology suggests that female-specific fronto-limbic/salience patterns associated with emotional processing may represent a risk factor for development of trauma-related pathologies of an affective nature (62). However, it should be underlined that in the current study we did not evaluate trauma-related symptomatology, so these considerations may not fully apply to our sample. These findings highlight the increased vulnerability of maltreated girls to internalizing symptoms, such as feelings of inadequacy and struggles in social relationships, which may be linked to their exposure to maltreatment.

Consistent with behavioral findings, recent literature has explored the role of specific inflammatory biomarkers, such as interleukin-6 (IL-6), in mediating the relationship between maltreatment and psychopathological symptoms, highlighting sex differences in the associations between IL-6, interpersonal difficulties, and somatic complaints. A recent study reported a stronger association between IL-6 and interpersonal difficulties in females, while in males, a slight increase in the link between IL-6 and somatic complaints was observed, with no significant relationship detected between IL-6 and interpersonal difficulties (63). This may suggest a biological basis for the connection between somatic complaints and interpersonal difficulties that we also observed in females.

Clinicians’ ratings did not reveal any differences between sexes among either children or adolescents. This could be due, at least in part, to the pervasive nature of maltreatment, which may overshadow the influence of gender on overall functioning.

Finally, we aimed to explore age group differences in adaptive skills. In line with the results from CBCL, we found that adolescents had poorer adaptive functioning than children in the social domain. The transition to adolescence represents a critical phase for maltreated children, as this developmental period is marked by heightened environmental demands for social competence. Adolescents are expected to navigate more complex social interactions and relationships, which can be particularly challenging for those with a history of maltreatment. This result aligns with literature documenting a relationship between emotional neglect and poorer relationship quality in adolescence (64). However, it should be emphasized that some studies have failed to observe such a relationship (64); these discrepancies may be attributed, at least in part, to differences about the participants involved—for instance, variations in the kind, timing, and severity of maltreatment.

Few sex differences in adaptive skills emerged among children, with females showing slightly better adaptive functioning in the social domain. Conversely, among adolescents, boys exhibited a significantly worse adaptive functioning, especially in the conceptual and practical domains. The poorer adaptive skills observed in males within the conceptual domain may be explained by the results of studies on sex differences in adolescents, based on parent reports, which identified more aggressive behaviors in boys. In fact, the composite score of the ABAS-II for the conceptual domain includes self-direction skills (e.g., ‘controls temper when disagreeing with friends’) that could be negatively affected by aggressiveness. The conceptual domain of the ABAS-II also includes items related to communication skills. Our results are consistent with previous research on the general population, which reports that females tend to outperform males in the area of communication skills (65, 66). Girls also exhibited better functioning than boys in the practical domain, suggesting they have better functioning in self-care, home/school living, community use, health and safety areas. Our results mirror previous findings on general population, indicating that, among children displaying below-average adaptive skills, females tend to exhibit better daily living skills (66).

Of note, no sex differences on social functioning emerged in adolescence, with both groups exhibiting low scores. These findings are partially inconsistent with previous reports documenting that boys who experienced sexual abuse in adolescence exhibit less companionship than did girls (67). A possible explanation for the discrepancy with previous literature may lie in methodological differences across studies, such as the data sources used (e.g., self-reports vs. parent reports), specific sample characteristics (e.g., age, type, and severity of maltreatment), or the particular domains of adaptive functioning assessed. Our results may suggest trajectories that, while diverging from a psychopathological standpoint, ultimately converge in a deterioration of social functioning during adolescence among maltreated youth. These difficulties may also root in alterations in dysfunctions of cognitive processing: indeed, previous studies indicated that, compared to their peers, maltreated children are less likely to perceive (68, 69). Our results, which suggest impairment in adaptive functioning in the social domain of maltreated children and adolescents, strongly emphasize the need for a more in-depth understanding of these deficits, as impaired social functioning during adolescence can further hinder their overall development and integration into society.

Grounded in psychological and developmental theory, it is plausible that maltreatment influences interpersonal functioning, as individuals tend to form attachment styles that are dysfunctional even outside of family dynamics (70). Moreover, our findings could be interpreted through the lens of Developmental Trauma theory, which posits that chronic exposure to early adversity disrupts normative developmental processes across emotional, cognitive, and social domains (71). According to this framework, early maltreatment can dysregulate biological stress systems, such as the hypothalamic-pituitary-adrenal axis, and alter neurodevelopmental trajectories, leading to sex-specific psychopathological outcomes (71, 72). Therefore, the observed sex differences in somatic complaints, aggressive behaviors, and internalizing symptoms align with the notion that potentially traumatic events affect boys and girls differently, partly due to differential biological vulnerabilities and emotional and behavioral responses. This theory also underscores the cumulative impact of trauma across developmental stages, which may explain why we observed greater social impairments in adolescents compared to children. However, our study did not include specific measures to assess trauma-related symptoms, so interpretations based on Developmental Trauma Theory should be approached with caution.

Altogether, the results of the current study seem to support the idea that youth with a history of maltreatment constitute a critically distinct subtype across adaptive profiles, internalizing symptoms and externalizing problems. This may align with the hypothesis of the existence of different “ecophenotypes” among maltreated individuals, conceived as a phenotypic specialization (phenocopy) resulting from environmental experience (19). However, further research is needed to fully establish these ecophenotypes and their underlying mechanisms.

The results of the current study should be interpreted in light of some limitations. The first concerns the cross-sectional design: exploring psychopathological trajectories in maltreated children would be more informative with a longitudinal approach. The second limitation is the absence of a more comprehensive self-report instrument to detect psychopathological symptoms as well as trauma-related symptoms. Future studies should incorporate additional self-report tools that capture both internalizing and externalizing issues. Another limitation is that, although it is acknowledged that adolescents may have been exposed to abuse for varying durations, the study did not explore the severity or type of abuse experienced, nor how the duration or intensity of abuse may affect boys and girls differently. These factors could significantly influence the results and limit the ability to generalize findings across different forms of maltreatment. Finally, although parent and adolescent reports were partially corroborated, the lack of an analysis of discrepancies between perspectives could limit the interpretation of the results.

Despite these limitations, the present research offers new insights into the clinical manifestations of psychopathological symptoms and on the adaptive functioning of children and adolescents who experienced maltreatment. Altogether, our results, supported by both parent- and self-reports, suggest that the social domain represents one of the most affected domain in the transition to adolescence of maltreated children. The early identification of social difficulties in maltreated children is crucial to prevent long-term consequences on their emotional well-being and interpersonal relationships. Implementing comprehensive assessment strategies that integrate both parent- and self-reports can enhance the accuracy of identifying specific areas of impairment and guide the development of personalized intervention plans. Our findings also underscore the need for targeted clinical interventions to address the worsening social adaptation in this population. Clinicians should prioritize developing strategies to enhance social skills and adaptive functioning in adolescents who have experienced maltreatment, as early and effective support can mitigate long-term negative outcomes and improve overall well-being. A key characteristic of most social skills training programs is their focus on developing, practicing, generalizing, and maintaining pro-social behaviors while reducing or eliminating conflicting problem behaviors (73). Therefore, according with the clinical needs, targeted interventions may prioritize some domain, such as interpersonal communication, assertiveness, problem solving, self-control and stress management (74). Additionally, promoting a supportive environment across different contexts, such as family, school, and community settings, may further facilitate the generalization and maintenance of newly acquired social skills, fostering more positive developmental trajectories for maltreated youth. Finally, the differential profiles of psychopathological symptoms in maltreated boys and girls highlights the need for gender-sensitive clinical approaches.

## Data Availability

The raw data supporting the conclusions of this article will be made available by the authors, without undue reservation.
